# Efficient collection of a large number of mutations by mutagenesis of DNA damage response defective animals

**DOI:** 10.1038/s41598-021-87226-7

**Published:** 2021-04-07

**Authors:** Yuji Suehiro, Sawako Yoshina, Tomoko Motohashi, Satoru Iwata, Katsufumi Dejima, Shohei Mitani

**Affiliations:** 1 Department of Physiology, Tokyo Women’s Medical University, Shinjuku, Tokyo Japan; 2 Tokyo Women’s Medical University Institute for Integrated Medical Sciences, Shinjuku, Tokyo Japan; 3 Chubu University Center for Education in Laboratory Animal Research, Kasugai, Aichi Japan

**Keywords:** Mutagenesis, Computer science

## Abstract

With the development of massive parallel sequencing technology, it has become easier to establish new model organisms that are ideally suited to the specific biological phenomena of interest. Considering the history of research using classical model organisms, we believe that the efficient construction and sharing of gene mutation libraries will facilitate the progress of studies using these new model organisms. Using *C. elegans*, we applied the TMP/UV mutagenesis method to animals lacking function in the DNA damage response genes *atm-1* and *xpc-1*. This method produces genetic mutations three times more efficiently than mutagenesis of wild-type animals. Furthermore, we confirmed that the use of next-generation sequencing and the elimination of false positives through machine learning could automate the process of mutation identification with an accuracy of over 95%. Eventually, we sequenced the whole genomes of 488 strains and isolated 981 novel mutations generated by the present method; these strains have been made available to anyone who wants to use them. Since the targeted DNA damage response genes are well conserved and the mutagens used in this study are also effective in a variety of species, we believe that our method is generally applicable to a wide range of animal species.

## Introduction

Model organisms such as nematodes, flies, and mice have traditionally been used for genetic research^1^. In recent years, advances in massive parallel sequencing technology have facilitated the sequencing of whole genomes and have led to the establishment of new model organisms that are more suitable as models for various biological phenomena. If a gene knockout mutant library could be easily built in these new model organisms, the research could be accelerated, similar to what has occurred in existing model organisms^2,3^. There are two main methods for generating mutants. One is to use genome-editing techniques to induce mutations in target genes^4–6^. The other is to introduce mutations randomly by chemicals or transposons. CRISPR and other genome-editing techniques are useful for adding designed mutations to genes of interest. However, this technique cannot be used to obtain mutants of genes that were not predicted to exist, and is expensive to construct thousands of mutants. On the other hand, random mutagenesis allows us to obtain a large number of nonbiased mutations at one time^7,8^. Although the identification of the mutations has been considered to be time-consuming, recent inexpensive whole-genome sequencing (WGS) is compensating for this shortcoming^9^.

*C.elegans* is one of the classic model animals, and many mutants have been generated by introducing random mutations. We previously isolated thousands of deletion mutants by trimethyl psoralen and ultraviolet (TMP/UV) mutagenesis^10–12^. Deletions introduced by TMP/UV that are tens of bases or more are easy to genotype and use for functional analysis. In addition, since the frequency of mutations per strain is low, individual mutations can be segregated by crossing and can be used for individual gene function analysis. However, due to the small number of mutations per strain, the efficiency of mutant library construction is low. Another group reported an alternative approach^13^ to introduce high-density point mutations and small indels per line using DNA alkylation agents such as EMS and ENU. However, it has been reported that point mutations rarely show a phenotype, except for those causing amino acid variations within important motifs^14^. Also, although high-frequency mutations are suitable for forward genetic screening, they are not a suitable approach for functional analysis of individual genes because of the effort required to isolate individual mutations.Therefore, to efficiently construct mutant libraries for the analysis of individual gene functions, it is important to establish methods for the generation of organisms with mutations that can be easily genotyped with high frequency to the extent that they can be isolated by crossing.

The TMP/UV mutagen induces DNA monoadducts and interstrand crosslinks (ICLs) and subsequent double-strand breaks (DSBs)^15–17^, causing deletion mutations, in a wide range of species^18,19^. When the genome of organisms is damaged by mutagens, the DNA damage response (DDR), such as DNA repair and signaling pathways for cell cycle control or apoptosis, is activated^20^. DNA monoadducts are repaired by two major nucleotide excision repair (NER) pathways, and ICLs are repaired by the Fanconi anemia pathway, NER, and homologous recombination (HR) ^21–23^. There are two types of DNA damage recognition mechanisms in the NER pathway, one is the global genome NER initiated by damage recognition by XPC, and the other is the transcription-coupled NER involving CSA and CSB^21,22^. All these repair pathways are conserved in *C.elegans* and are activated by TMP/UV mutagenesis^17^. DSBs can be repaired through two major classically studied pathways, nonhomologous end joining (NHEJ) and HR, or other pathways, such as microhomology-mediated end joining (MMEJ)^24,25^. DSBs activate not only the repair but also the cell cycle control and the apoptosis pathways driven by ATM and other factors^26^. Since the loss of these DDR-related factors is considered to lead to the accumulation of mutations and subsequent carcinogenesis and aging^20^, we treated DDR-deficient mutants with TMP/UV as a new mutagenesis strategy for introducing a "moderate" number of mutations. Here, we report that TMP/UV treatment of XPC and ATM double mutants and subsequent bioinformatics analysis by WGS and machine learning allows us to isolate new mutant lines three times more efficiently than mutagenesis of wild-type animals. As XPC and ATM are well-conserved genes, and TMP/UV has effects even on mammalian cells^18^, our mutagenesis method is expected to apply to other species. Using this method, we isolated 981 new mutant strains, which we are distributing to the research community.

## Results

### Accumulation of novel variants in the DDR mutant strains

**Figure 1:**
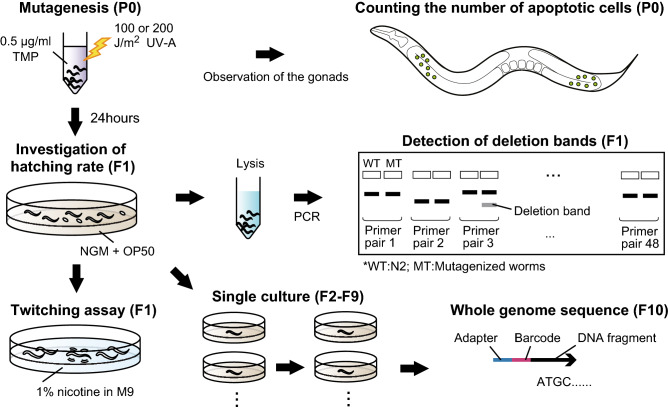
Experimental design. The adult worms were exposed to TMP and irradiated with UV. Apoptotic cells in the gonads were counted 2 h after and obtained F1 eggs by washing off the adults and larvae 24 h after the mutagenesis. The hatching rates were measured the next day. The thousands of the siblings of hatched F1 larvae were soaked with 1% nicotine for the twitching assay and other thousands of F1 worms were lysed to detect deletions by PCR. For the whole genome sequencing, more than 5 F1 worms were randomly picked and cultured individually. The single culturing was repeated until the F9 generation and the starved F10 worms were used to extract genomic DNA. The extracted genome was sheared and about 120 base fragments were tagged by the adapter and barcode sequence. Finally, the mixture of the tagged DNA was sequenced using ionProton system.

Genomic DNA damage is repaired by the DDR system. Since the loss of function of DDR-related genes leads to the accumulation of mutations in *C. elegans*^27^, we hypothesized that the TMP/UV treatment of DDR mutants leads to efficient isolation of mutant strains. To test this hypothesis, among the genes involved in the response to monoadducts, ICLs, and DSBs^28–34^, mutants of the following seven genes, which have already been isolated in our laboratory (Supplementary Table S1), were treated with TMP/UV: *atm-1*, *wrn-1*, *ced-4*, *cku-80*, *polq-1*, *pcn-1*, and *xpc-1* (see Supplementary Fig. S1). Although the *pcn-1* mutant allele used in this research does not lack coding regions, we confirmed that the expression level of *pcn-1* decreased in the mutant (Supplementary Fig. S2). After the mutagenesis, we performed a twitching assay^35^ using F1 larvae to evaluate the mutation frequencies and estimate the number of additive mutations per strain (Fig. 1). The mutation frequency in the *polq-1* mutant was larger than that in the wild type, but other DDR mutants showed the same or even smaller number of variants than the wild type (Table 1). In the twitching assay, however, mutations with no behavioral phenotype may be missed. Therefore, we further performed PCR on 48 sites of chromosome III (Supplementary Table S2) to search for novel deletions. This analysis confirmed additive deletion in the DDR mutants except for *wrn-1*, *polq-1*, and *atm-1* (Table 1). The *polq-1* mutant showed conflicting results in the twitching assay and PCR detection. Since other groups have reported that *polq-1* mutants generate deletions larger than 10 kb^32^, the mutations in *polq-1* might have been too large to detect by PCR. Overall, these results indicate that the DDR mutants, except for *wrn-1* and *atm-1* mutants, can accumulate small mutations or deletions at a higher frequency than the wild type under our mutagenesis conditions.

**Table 1:** Forward mutation frequencies and survival rates of F1 worms after the TMP/UV mutagenesis.

Strain	Hatching rate (%)	Twitching assay		PCR detection	
		Mutation frequency (%)	Estimated number of mutations	Mutation frequency (%)	Estimated number of deletions
N2	100	0.22	10.2	< 0.05	0.00
*wrn-1*	100	0.04	1.7	< 0.05	0.00
*ced-4*	90	0.14	6.5	0.1	1.12
*cku-80*	95	0.07	3.3	0.2	2.23
*polq-1*	95	0.60	28.0	< 0.05	0.00
*pcn-1*	100	0.06	3.0	0.1	1.12
*atm-1*	90	0.10	4.7	< 0.05	0.00
*xpc-1*	20	0.23	10.6	0.05	0.56
*atm-1;xpc-1*	40	0.09	4.2	0.025	0.28

Forward mutation frequencies were calculated by two methods, twitching assay and PCR detection. The estimated numbers of variants were calculated according to the equations described in materials and methods.

### Survival rates of TMP/UV-treated DDR mutants

**Figure 2:**
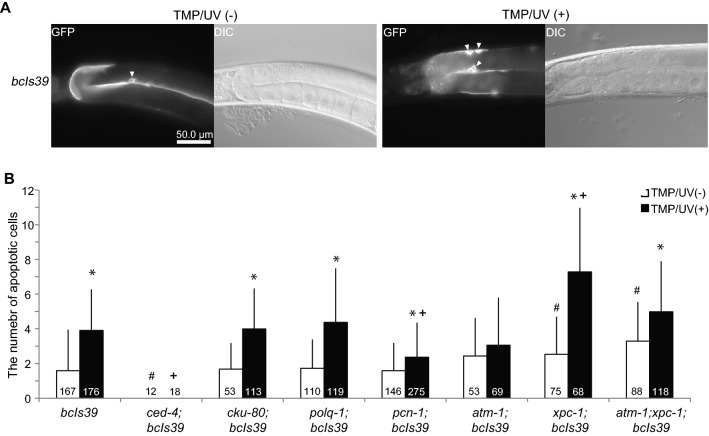
Germ cell apoptosis in the TMP/UV treated DDR mutants. (**A**) Representative images of intact and TMP/UV treated *bcls39 [CED-1::GFP]*. White arrowheads indicate apoptotic cells. (**B**) The averaged numbers of apoptotic germ cells two hours after TMP/UV mutagenesis were shown as a bar graph. The white bars demonstrate the non-treated worms and the black bars indicate the result of TMP/UV treated worms. The error bars demonstrate the SD. All the data were tested by Steel Dwass’s Test using R software^56^. *p < 0.05, differences between TMP/UV(–) and TMP/UV(+) of each strain, #p < 0.05, differences between TMP/UV(−) mutants and TMP/UV(−) wild-type, + p < 0.05, difference between TMP/UV(+) mutant and TMP/UV(+) wild-type. The p-values between other pairs are summarized in Supplementary Table S3. The numbers of observed gonads are displayed on each bar.

As shown in Table 1, estimated numbers of deletions in *wrn-1* and *atm-1* genes were very low. As these genes are not directly involved in the DNA repair, DNA damages may be repaired in mutants of the genes. Alternatively, the mutated cells in the mutants may be eliminated before they could develop. In *C. elegans*, germ cell apoptosis occurs in pachythene stage meiotic cells^36^ and the apoptosis is induced by the accumulation of DNA damages^37,38^. To investigate whether germ cells were excessively eliminated in the DDR mutants by the apoptosis, we visualized apoptotic cells in the gonads^35^ and quantified cell death. Under our mutagenesis conditions, the number of apoptotic cells in wild-type animals was increased by approximately two-fold after TMP/UV treatment (Fig. 2A). No apoptosis was observed in *ced-4* mutant, whether treated or untreated with TMP/UV (Fig. 2B, Supplementary Fig. S3). Since it has been reported that *ced-4* is a major factor in initiating apoptosis^30^, it is considered that mutated cells were not removed by apoptosis in *ced-4* mutant. The *xpc-1* and *atm-1* genes were reported to regulate UV mediated DNA damage-dependent apoptosis positively^39^. We found that the number of apoptotic cells in *atm-1* was not increased by TMP/UV treatment (Fig. 2B, Supplementary Fig. S3), indicating that *atm-1* is also required for germ cell apoptosis after TMP/UV treatment. However, in our results, TMP/UV treatment significantly increased apoptosis in the *xpc-1* mutant (p-value is 6.0E−13). In addition, the number of apoptotic cells in TMP/UV treated *xpc-1* was significantly higher than TMP/UV treated wild type (p-value is 2.1E−10, Supplementary Table S3). These data are inconsistent with the report of another group using UV-C as mutagen^39^, suggesting that another apoptosis pathway independent on *xpc-1* is dominant during the TMP/UV-induced damage response. Our data also showed suppression of apoptosis in the *pcn-1* mutant.

Even if germ cells carrying mutations escape apoptosis in the gonads, eggs with accumulated mutations are reported to cause the failure of hatching in *C.elegans*^40^. Then, we examined the hatching rate of the DDR mutants after TMP/UV treatment. The *xpc-1* mutant showed a decrease in hatching rate, but no other mutants showed an extreme decrease (Table 1). The result in *xpc-1* mutant is consistent with the report that deficiency of *xpc-1* and some other ICL repair factors reduce embryonic survival after TMP/UV treatment^17^. Given these results, we propose that the increased apoptosis or the failure of hatching in the *xpc-1* mutant may have reduced the number of living F1 larvae carrying TMP/UV induced mutations. To test this idea, we inhibited the apoptosis and the hatching failure in *xpc-1* by crossing with *atm-1* mutant. It has been reported that the loss-of-function of *xpa-1* increases germ cell apoptosis without mutagenesis and that the mutagen independent apoptosis in *xpa-1* mutant is mitigated by the loss of function of *atm-1*^39^. As *xpa-1* works in concert with *xpc-1*^23^ we hypothesized that the loss of *atm-1* function mitigates the low survival rate of *xpc-1* mutants after TMP/UV treatment. As we expected, apoptosis rates were rescued in *atm-1;xpc-1* compared to the *xpc-1* single mutant after TMP/UV treatment (p-value is 0.00062, Supplementary Table S3) and the hatching rate in *atm-1;xpc-1* tended to be higher than *xpc-1* mutant (Table 1). Then, we examined the mutation frequency of the TMP/UV-treated *atm-1;xpc-1* double mutant by twitching assay and PCR. However, the mutation frequencies by both methods were not increased rather than *xpc-1* mutant (Table 1). Thus, we considered that the rescue of survival rate would not induce the increase of mutation frequency in F1 larvae.

### Detection of novel variants in TMP/UV-treated DDR mutants

Some of the mutants generated by TMP/UV treatment exhibit phenotypes such as growth abnormalities, lethality, or sterility and thus cannot be acquired as viable strains. To investigate how many novel mutant strains could be isolated from each DDR mutant, we cultured mutagenized F1 larvae for 10 generations (Fig. 1) and detected the homozygous variants from the established lines by WGS. The sequencing conditions and accession numbers of the data are shown in Supplementary Table S4. The detected variants were classified into two groups. The first was small variants, which were detected by the VariantCaller (Thermo Fisher Scientific) and were less than 20 bases. The other was the large variant, which contains deletions of more than 50 bases, detected by our program (see Supplementary Document and Supplementary Fig. S4). Since the larger variants are easy to detect by PCR, we confirmed the breakpoints of large variants by Sanger sequencing and mainly analyzed them in this study (Supplementary Table S5). Despite the high mutation frequency in F1 larvae (Table 1), no homozygous large variants were obtained from *polq-1* mutant-derived lines. As the variants showing recessive lethality cannot be isolated as homozygotes, we searched for heterozygous variants from *polq-1* mutants. Then, as we found one heterozygous variant, we included it in the subsequent analysis.

**Figure 3:**
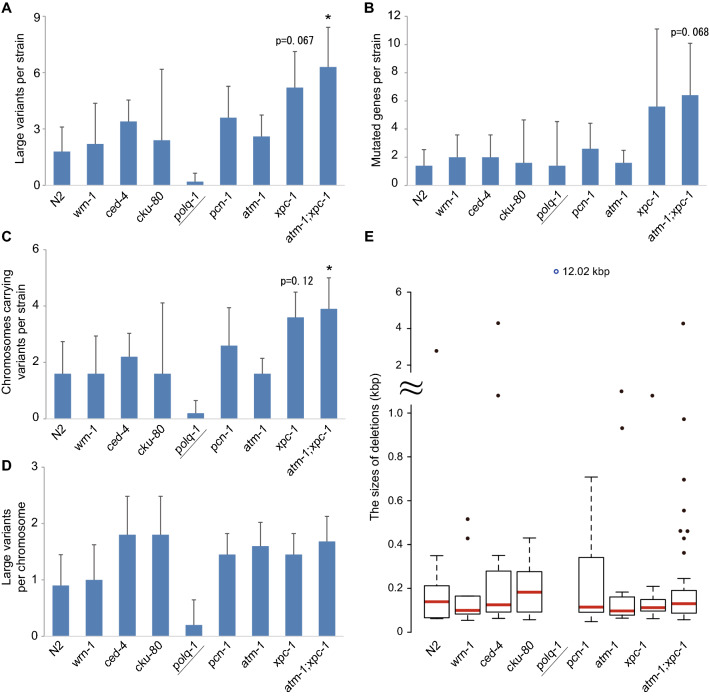
Whole genome sequencing of DDR mutants. The 5 or 10 independent lines of the TMP/UV treated N2 and DDR mutants were clonally propagated for 10 generations and the whole genome of the worms was sequenced using ionProton (Thermo Fisher Scientific). (**A**) The number of deletions larger than 50 bp for each strain is shown in a bar graph. Not only simple deletions but also complex deletions with insertions or inversions were counted. The *polq-1* mutant shows heterozygous mutations, while the others are homozygous variants. The *atm-1;xpc-1* mutant showed a significantly larger number of variants than wild-type by Steel’s test (p-value is 0.034, marked as asterisk). (**B**) The number of genes that were partially or completely deleted for each strain is shown in the bar graph. The *atm-1;xpc-1* mutant showed not significant but the highest values (p-value is 0.068, Steel’s test). (**C**) The number of chromosomes carrying large variants per strain is shown in the bar graph. The *atm-1;xpc-1* mutant showed a significantly larger value than wild-type by Steel’s test (p-value is 0.036, marked as asterisk). (**D**) The number of large variants per chromosome was shown in the bar graph. There was no significant difference between wild-type and DDR mutants by Steel’s test. (**A**–**D**) The error bars mean the SD. (**E**) The distribution of the deletion sizes (Supplementary Table S5). The upper panel shows larger than 1 kb and the lower panel shows less than 1 kb deletions. The variant derived from *polq-1* mutant is shown as a labeled blue circle because the size of it was extremely outside the range of the other variants. The red lines and boxes indicate the median values and interquartile ranges, respectively. The whisker shows a range of 1.5 × interquartile range. There was no difference among the strains by Kruskal–Wallis rank sum test (p-value is 0.63). All statistical analyses were performed using R software^56^.

Among the DDR mutants tested in this study, the number of large variants was threefold higher in the *xpc-1* and *atm-1;xpc-1* mutant-derived strains than in the wild type, and twofold higher in the *ced-4* and *pcn-1* mutant-derived strains. In particular, the largest number of large variants was isolated from the *atm-1;xpc-1 I*-derived strains, which were significantly more than the wild type (p-value is 0.034, Fig. 3A). The frequency of detection of small variants was also significantly higher in lines derived from *atm-1;xpc-1* mutants than in wild-type (Supplementary Fig. S5, Supplementary Table S6). Next, we examined whether the obtained variants affect gene function and counted the number of genes that overlapped with the variants. Then, we found that the *xpc-1* and *atm-1;xpc-1* mutants showed not significant but larger numbers of gene structure changes than the wild-type (p-values are 0.068 and 0.27, respectively. Figure 3B). These results indicate that TMP/UV treatment of worms with mutations in *xpc-1* can introduce large varaints with higher efficiency than the wild type. In constructing a mutant library, the higher the expected cumulative number of mutations obtained from each P0 worm, the more efficiently mutant strains can be isolated. The survival rate of F1 larvae was higher in the *atm-1;xpc-1* double mutant than in the *xpc-1* single mutant (Table 1), suggesting that TMP/UV treatment of *atm-1;xpc-1* is expected to be more efficient.

Germline apoptosis after TMP/UV treatment was lower in *ced-4* and *pcn-1* mutants (Fig. 2B), which had more large mutations than the wild type, suggesting that the inhibition of germline apoptosis may increase the efficiency of mutation isolation by reducing the removal of mutated cells. The increased total number of both large (Fig. 3A) and small variants (Supplementary Fig. S5) detected from *atm-1;xpc-1* compared to *xpc-1* mutants also supports this possibility. In *xpc-1* mutant, however, the number of variants has increased despite increased apoptosis compared to the wild-type (Fig. 2B). This may be due to the accumulation of mutations caused by the inability to repair monoadducts and/or ICLs beyond their removal by apoptosis. The WGS results differed from the F1 mutation frequencies in two points: first, the mutation frequency of *atm-1;xpc-1* was not elevated compared to *xpc-1* in F1 larvae; second, the number of variants detected by WGS was higher than that estimated in the F1 generation (Table 1). One possible reason is that new variants have been introduced during culturing. It has been reported that spontaneous mutation from G/C to A/T nucleotides occurs preferentially in *C. elegans*^41^. Then, we investigated the patterns of nucleotide substitution of the small variants (Supplementary Table S6) and found that the major substitution was from A/T to T/A in the wild-type and DDR mutants except for *polq-1* (Fig. S5). The frequent substitution of A/T with T/A may be due to the chemical mechanism of TMP/UV leading to the generation of monoadducts or crosslinks at 5′-TA dinucleotides^15^. We further performed WGS of the F4-F5 generation derived from TMP/UV-treated *atm-1;xpc-1* and searched for hetero- and homozygous variants to test whether the number of large variants is different between generations. Then, we found that the total number of heterozygous and homozygous variants detected in the F4-F5 generations was not different from the number of homozygous variants obtained in the F10 generation (Supplementary Fig. S6). From these data, we considered that variants detected by WGS to be mainly TMP/UV-dependent mutations at P0 rather than spontaneous mutations, and that non-phenotypic mutations or large size mutations were missed in F1 larvae.

### Distribution of variants in TMP/UV-treated DDR mutants

The more mutations per strain, the more mutant lines can be isolated, but the more time it takes to isolate them. In particular, as the number of mutations per chromosome increases, the effort to isolate them increases, because chromosomal recombination must have occurred at the right locations. To explore the distribution of variants in each chromosome among the DDR, we counted the number of chromosomes with variants per strain (Fig. 3C) and the variants per chromosome (Fig. 3D). In the *xpc-1* and *atm-1;xpc-1* mutant, the average number of chromosomes carrying deletions per strain was larger than three (Fig. 3C), and the difference between wild-type and *atm-1;xpc-1* was significant (p-value is 0.036). On the other hand, the average number of variants per chromosome was not significantly different among all strains, and the values in *xpc-1* and *atm-1;xpc-1* were less than two (Fig. 3D). Since *C. elegans* has six chromosomes and approximately half of them are transmitted from F1 to F10 according to Mendel's law, the TMP/UV treatment of *xpc-1* and *atm-1;xpc-1* mutants is considered to reduce the cost of isolation of mutations on the same chromosome while increasing the number of novel mutations per strain.

### The size distribution of deletions induced by DSBs in DDR mutants

As variants larger than a few dozen kilobases can affect multiple genes (Fig. 3B), the size of the mutation is potentially related to the quality of the mutant library. To evaluate the effect of DDR mutants on the size of mutations, we compared the size distribution of detected variants. The median sizes of the variants were approximately 100–200 bases in all DDR strains and the values were not different from the wild-type (Fig. 3E). As the median gene size in *C. elegans* is 1,956 bases^42^, each variant obtained from DDR mutants except *polq-1* can be expected to affect mainly a single gene.

**Figure 4:**
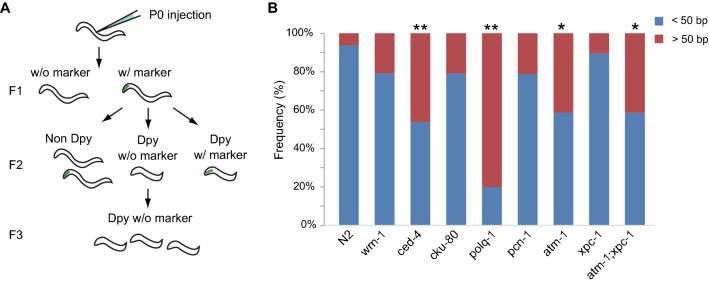
The size distribution of CRISPR/Cas9-mediated deletions. (**A**) Overview of experimental design. Constructs for Cas-9 mediated genome editing were injected into the gonad of adult worms. Then, the F1 larvae carrying transgene were selected. From F2 progeny, we picked larvae, which show the dumpy phenotype but not the transgenic marker to eliminate worms carrying extrachromosomal array of Cas9 gene. After the confirmation that all the F3 worms showed the dumpy phenotype, the deletion sizes were confirmed by Sanger sequence. Primers used for the sequencing were designed to cover the regions from 32 kb upstream to 41 kb downstream of the *dpy-3* gene (Supplementary Table S7). (**B**) The size of deletions was classified into two groups, larger than 50 bp (red) and less than 50 bp (blue). Then, the relative frequencies of each group were displayed as a stacked bar chart. **p < 0.01, *p < 0.05, Fisher's Exact Test. All statistical analyses were performed using R software^56^.

The TMP/UV method can also introduce variants smaller than 50 bases, as confirmed by WGS results (Supplementary Figure S5). Although these small variants can affect gene function, they were not targeted for analysis. Then, we next examined what percentage of these small variants are generated in wild-type and DDR mutants. Since the WGS analysis, however, can miss false-negative variants, we used the CRISPR/Cas9 system to directly induce DSBs in the *C.elegans* genome and then compared the size distribution of the deletions formed. Targeting the *dpy-3* gene, we collected worms with mutation based on their phenotype^13^ and determined the deletion sizes by Sanger sequencing (Fig. 4A, Supplementary Table S7). The results showed that 93.8% of the deletions were smaller than 50 bases in the wild-type, and this percentage was significantly lower in *ced-4*, *polq-1, atm-1,* and *atm-1;xpc-1* (Supplementary Table S8, Fig. 4B, p-values are 0.0073, 9.51E-06, 0.039, and 0.016, respectively, Fisher’s exact test). The data that the loss of function of *polq-1* leads to large deletion formation was consistent with another group’s study^32^. In addition, the result suggests that *ced-4*, *atm-1*, and *atm-1;xpc-1* mutants are prone to larger deletions, and if a DSB occurs randomly in a gene, the probability of missing important domains by larger mutation may increase.

### Large-scale whole-genome sequencing

**Figure 5:**
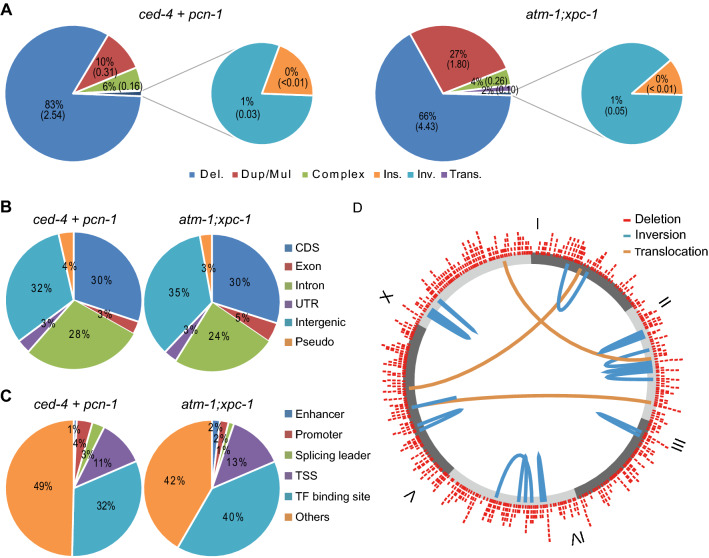
Large scaled NGS. The whole genome of 10, 124, and 306 strains derived from *pcn-1*, *ced-4*, and *atm-1;xpc-1* were sequenced, and homozygous variants were detected, respectively. (**A**) The colored pie graph shows the frequency of variants. Deletions (blue), duplications and multiplications (red), and complex deletion with insertion (green), and insertion (violet) are shown in large pie graphs, and inversions (cyan), and translocations (orange) were shown in small pie graphs. The distribution of each type of variants were different between *atm-1;xpc-1* and the group of *ced-4* and *pcn-1* (p-value is 1.7e-4, chi-square test). (**B**) The variants were annotated and classified into the 6 types, CDS (blue), exon (red), intron (green), UTR (violet), intergenic region (cyan), and pseudogene (orange). There was no difference among the strains (p-value is 0.38, chi-square test). (**C**) The variants annotated as intergenic regions in (**B**) were classified into the following 6 types, enhancer (blue), promoter (red), splicing leader (green), transcription start site (TSS, violet), transcription factor (TF) binding site (cyan), and the others (orange). There was no difference among the strains (p-value is 0.18, chi-square test). (**B**,**C**) For the annotation, we used datasets obtained from WormBsae^43^ (WS252, ftp://ftp.wormbase.org/pub/wormbase/). (**D**) The positions of deletions (red) and rearranged variants were shown as a circular diagram using our script (https://github.com/YujiSue/RScript/blob/master/roundGraph.R). The break sites of 16 inversions (cyan) and 3 translocations (orange) detected from *ced-4* and *atm-1;xpc-1* (Supplementary Tables S5, S9) were joined by the colored curves in the circle. All statistical analyses were performed using R software^56^.

To investigate the detailed properties of variants obtained by our method, we sequenced an additional 299 mutagen-treated lines. For the analysis, we used *ced-4*, *pcn-1*, and *atm-1;xpc-1*, because of the high frequency of mutations obtained in these mutants (Supplementary Table S4). As the mutagenized *xpc-1* showed a higher mortality rate, we did not analyze the *xpc-1* mutant. The average number of deletions per strain was larger in *atm-1;xpc-1* than *pcn-1* and *ced-4* (Fig. 5A, Supplementary Table S9) as shown in the small-scaled sequence (Fig. 3A). In addition, frequent duplications or multiplications were shown in *atm-1;xpc-1* rather than *ced-4* and *pcn-1*, and structural variants such as inversions and translocations were detected at a frequency of approximately 1% in both groups.

Next, we annotated the variants with deletions and found that approximately two-thirds resulted in the deficiency of genes or pseudogenes, regardless of background genotype. Approximately 30% of these gene-affecting mutants had a partial or complete deletion of the CDS of at least one gene (Fig. 5B). This frequency is higher than the value of approximately 8% reported by other groups that introduced hundreds of small mutations per strain^14^, suggesting that the introduction of deletion variants by TMP/UV more efficiently produced mutations affecting the protein-coding genes. Furthermore, we also annotated the variants in intergenic regions, and we found that more than half of them were located in areas annotated with enhancers, promoters, and transcription factor binding regions^43^ that could be involved in transcriptional regulation (Fig. 5C). Therefore, more than 80% of the detected variants were found to be usable for gene function analysis even when the variants were derived from the *ced-4*, *pcn-1*, and *atm-1;xpc-1* mutants in *C. elegans*.

Even when the obtained variants lack gene function, it is not useful for building a mutant library if they cause mutations with a bias towards only certain genes. As TMP/UV treatment generally induces mutations at AT sites^15^, we investigated the correlation between the locations of isolated variants and the density of A or T bases per 100 kb. There was no correlation between mutation site and AT content (R^2^ is 0.0049, Supplementary Fig. S7), and the locations of variants were spread across the whole genome (Fig. 5D). These results indicate that TMP/UV mutagenesis using *atm-1;xpc-1* can induce gene mutations in a highly efficient and random manner.

### Selection of candidates by machine learning

**Figure 6:**
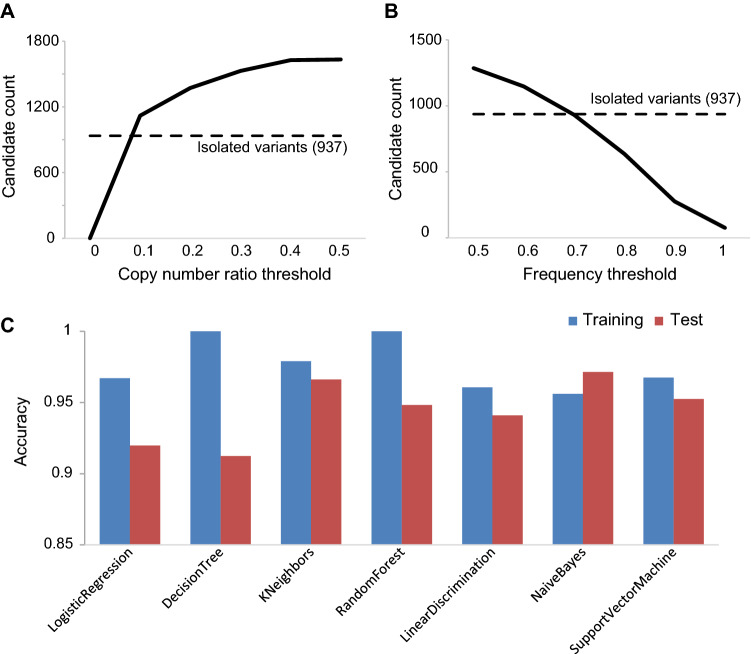
Machine learning to filter false-positive candidates. (**A**,**B**) In the general copy number analysis of WGS data, the copy number ratio value (Supplementary Fig. S4) is used for indicator of deletions, and the frequency of a variant is used for the criterion whether it is homozygous or not. From the detected candidate variants, we counted the number of them with lower than a certain value of the normalized depth (**A**), and the number of them with higher than a certain value of the frequency (**B**). The broken line shows the number of isolated homozygous variants. (**C**) For the selection of variants by machine learning, we tested 7 algorithms, logistic regression (LR), decision tree (DT), k-nearest neighbor (kNN), random forest (RF), linear discrimination (LDA), naïve Bayes (NB), and support vector machine (SVM). We used the data listed in Supplementary Table S5 for the training and Table S9 for the test. After the training, we checked the accuracy of prediction whether the candidate is true or false using each learned model. The result of prediction to training data (blue bar) and test data (red bar) was shown. The analysis was performed using our original script (https://github.com/YujiSue/python/blob/master/DeletionFilter.ipynb).

As the number of candidate variants increases, the number of false positives also increases in bioinformatics analysis. However, since PCR genotyping is time-consuming and costly, the elimination of false positives by computer analysis is required for the efficient isolation of mutations. In the WGS analysis, since the number of false-positive candidates varies according to the analysis parameters (Fig. 6A,B), filtering by certain parameters risks eliminating true variants or retaining false positives. In recent years, machine learning has been used for sorting these variants. Therefore, we tested the following eight algorithms that have used filtering small variants: logistic regression (LR)^44^, decision tree (DT)^45^, k-nearest neighbor (kNN)^44,45^, random forest (RF)^44,45^, linear discriminant analysis (LDA)^45^, naïve Bayes (NB)^44,45^, and support vector machine (SVM)^44^. We then used these scores to determine if the target candidate was a true or false positive. For the training, we used data on homozygous deletions from DDR mutants (Supplementary Table S5). To test the performance of the trained model, predictions were made on the trained data, and all algorithms showed approximately 95–100% accuracy. Using this model, we made predictions on the results of large-scale sequencing (Supplementary Table S9) and found that k-NN, NB, and SVM showed more than 95% correct responses (Fig. 6C). Other algorithms also showed more than 90% correct response rates. Therefore, we conclude that the machine learning algorithms, especially k-NN, NB, and SVM under our conditions, are useful for the selection of true variants.

## Discussion

In this study, we have shown that we could isolate more mutations by TMP/UV-treatment of *xpc-1* or *atm-1;xpc-1* mutants than the wild-type. In particular, from the *atm-1;xpc-1* double mutant, we were able to isolate the highest number of mutations, approximately three times that of the wild-type (Fig. 3A). Although there are mutagenic chemicals that can introduce mutations at a higher frequency than TMP/UV, the high number of mutations per strain causes experimental limitations that require costs to isolate individual mutations. We believe that the mutant isolation by our method avoids the shortcomings by introducing mutations of "reasonable" size and "reasonable" frequency, and will improve the construction of mutation libraries for individual gene function analysis.

The TMP/UV causes monoadducts, ICLs, and the subsequent repair process results in DSBs. Then, during the pathway through *polq-1* for the DSBs repair, some errors result in deletions^42^. Since XPC is related to the initiation of response to the damages by TMP/UV^17^ and ATM is required for the response to DSBs and regulation of the apoptosis, the accumulation of mutations due to reduced repair of DNA damages and the survival of the mutated cells in *atm-1;xpc-1* mutants may have resulted in more efficient mutagenesis than the wild-type. The present study does not clearly show whether XPC contributed more to monoadducts or ICLs repair. However, in the DNA replication-dependent ICL repair pathway, such as in germ cells, it is generally known that Fanconi-Anemia-pathway acts more upstream. Considering that apoptosis induction occurred even in the absence of XPC (Fig. 2), defective repair of the monoadducts in *xpc-1* mutant contributed to the accumulation of mutations.

Many of the DDR factors, including ATM and XPC, are conserved in a wide range of species ^20,21,26^, and TMP has been reported to act on vertebrate and mammalian cells^10,18,19^. Therefore, it should be possible to efficiently isolate mutants in any species carrying a combination of deficiencies in major DNA repair and major DDR signaling pathways. The WGS results suggest that loss of function in XPC is sufficient to isolate mutants at a higher rate. However, it will be essential to reduce the mortality caused by the mutagenesis of XPC mutants when isolating a large number of mutants, especially in species with low reproductive rates or small litters, as we have overcome by using the *atm-1*;*xpc-1* double mutant.

Compared to *xpc-1* mutants, neither mutants of *cku-80*, a major factor in NHEJ, nor *polq-1*, which is involved in MMEJ, showed any effect on the efficiency of additive mutation isolation. *wrn-1* and *atm-1* single mutant, which are activated by DSBs, also showed no effect. Surprisingly, the accumulation of small variants was not found in *atm-1* (Supplementary Fig. S5). In general, it is considered that the loss of ATM function causes the accumulation of variants^46^. However, some studies have shown that the survival rate after mutagen treatment in ATM-deficient mice varies depending on the background strain^47^. Those research findings and our results suggest that the combined deficiency of ATM and other DNA damage response factors, such as XPC, may result in a synergistic effect. In addition to XPC, the other NER factors such as CSA and CSB, HR, and Fanconi anemia pathway-related factors are also involved in monoadducts and ICLs repair after the TMP/UV mutagenesis^17,20,21^. However, most of the mutants of the major factors of HR are lethal and were not tested in the present study. Some kinases, such as ATR, are reported to have functional overlap with ATM ^25,26^. Another combination of these factors may allow us to obtain mutations with higher efficiency than the combination of ATM and XPC. Testing such combinations will be a challenge in the future. It should also be noted that the approximately 100 Mb genome of *C. elegans* is relatively small, especially when compared to the genomes of vertebrate model species. The deletion size we obtained in this research (Fig. 3E) may be too small to generate gene deletion in species with introns and intergenic regions exceeding a few kilobases. In *C. elegans*, however, loss of function of POLΘ results in deletions of tens of kilobases after mutagenesis^32^. Therefore, in species with large genome sizes, POLΘ mutations, in addition to mutations in ATM and XPC, may be required for effective isolation of genetic mutations.

The Cas9-induced deletion sizes showed that almost all mutations formed during the DSB repair pathway are smaller than 50 bases in wild-type and that the deficiency of *ced-4* or *atm-1* increased the frequency of large deletions compared to the wild-type (Fig. 4B). Since DNA damage caused by both TMP/UV and Cas9 has been reported to form deletions during *polq-1*-mediated repair pathway in *C. elegans* and large deletions appear when *polq-1* functions are lost^32,48^, *ced-4* and *atm-1* may affect the pathways, altering mutation size. Different from DNA cleavage at a limited location by Cas9, since the mutation size can be affected by multiple factors, including the location and timing of the DNA damage, in random mutagenesis, the size distribution of mutations induced by TMP/UV and Cas9 do not always coincide. However, if *atm-1* and *ced-4* generally modulate the size of mutation formed by DSB repair errors, these factors may increase, at least in part, the larger mutations induced by TMP/UV.

From the large-scale sequencing, we isolated inversions and translocations (Supplementary Table S9). These chromosomal structural variants are not suitable for functional analysis of genes but are useful as balancer chromosomes to maintain lethal mutations^49,50^. The balancer chromosomes have the potential side benefit to isolate mutations of essential genes that cannot be maintained as homozygous mutants. We further investigated whether machine learning was applicable to accurately detect stable gene mutations and rare structural variants. Machine learning techniques have already been used to efficiently detect small mutations^44,45,51^. In this case, we used non-deep learning methods because we started with a small data set, and we found that k-NN clustering, naïve Bayes, and linear support vector machine algorithms showed a correct identification rate of 95% or more. Although the optimal algorithm and parameters may vary depending on the sequence conditions and data quality, judgment by machine learning is expected to play a role in the automatic selection of true variants even when shallower sequencing for reducing costs of WGS in organisms with large genome sizes.

Many species have not yet been established as model organisms but show important phenotypes to be researched. The efficient construction of gene mutation libraries in these organisms will accelerate biological and medical research by facilitating reverse genetic analysis. Also, once mutation libraries are constructed, it will be possible to use them as the basis for forward genetic approaches^52^. All the genes, drugs, and techniques used in this study can be applied to other species. Even if there are no XPC or ATM loss-of-function mutants, it will be possible to generate by CRISPR or other genome editing techniques^4–6^. Alternatively, even species that are less sensitive to TMP/UV, other drug options are known to induce DNA monoadducts and ICLs in a wide range of species, including nitrogen mustard compounds and platinum drugs^27^. For these reasons, we expect that our method will expand the possibilities of genetic analysis in many species.

## Materials and methods

### Strains and maintenance of worms

Worms were grown at 20 °C under standard conditions^53^. The strains used in this study are listed in Supplementary Table S1. All mutant strains were backcrossed at least twice.

### TMP/UV mutagenesis and analysis of F1 phenotypes

TMP/UV mutagenesis was performed as previously reported^10^. The worms were treated with 0.5 µg/ml TMP for an hour and irradiated with 100 or 200 J/m^2^ UV-A (Fig. 1, Supplementary Table S4). One day after UV radiation, the adult P0 worms and F1 larvae were washed away, and the retained eggs were incubated for another day. Then, the hatching rate was calculated based on the number of F1 living larvae and the total number of eggs on the plates. For the investigation of mutation frequencies, thousands of hatched larvae were soaked in 1% nicotine, and the number of twitching worms was counted. Since the twitching phenotype is caused by mutation of the *unc-22* gene^35^, the number of mutations per haploid was estimated with the following equation.

$$Number \,of \,mutations=Twitching \,rate* \frac{100272607 (\mathrm{genome \,size})}{21477 (\mathrm{coding \,region \,of \,unc-22})}$$

Other larvae were divided into sets of 200 animals and lysed for the amplification of parts of chromosome III by stringent PCR. Then, the fragments that were uniquely detected in the mutagen-treated DDR mutants were extracted and sequenced by Sanger sequencing to confirm that the fragments contained deletions. The 48 primer pairs used for PCR were designed to cover 89,840 bases across the whole genome; their sequences are listed in Supplementary Table S2. The number of deletions was estimated with the following equation.

$$Number \,of \,deletions=Deletion \,rate* \frac{100272607 (\mathrm{genome \,size})}{89840 (\mathrm{total \,amplified \,regions})}$$

The total numbers of larvae used for the twitching assay and PCR detection are given in Supplementary Table S1.

### Observation of apoptotic cells in gonads

The *bcIs39* transgenic strain^38^ was crossed with our N2 and DDR mutant strains. Two hours after TMP/UV (200 J) mutagenesis, performed according to the method described above, the mutagenized and nontreated worms were paralyzed with sodium azide and placed on agarose pads. Then, apoptotic germ cells expressing *gfp* were counted under an Olympus BX-50 microscope.

### Whole-genome sequencing

TMP/UV-treated F1 worms were picked individually and transferred to a new plate for incubation. Every few days, grown adult worms were randomly picked and transferred to a new plate. We repeated this process up to the F9 generation. Then, after the F9 and F10 worms were starved, we collected them in M9 buffer and extracted their genomic DNA. Genomic DNA was isolated using a DNeasy Blood & Tissue Kit (Qiagen), and a DNA library was prepared from genomic DNA with a LibraryBuilder automatic library synthesis machine (Thermo Fisher Scientific) according to a customized protocol (details are described in the supplementary documents). The DNA library was used for the construction of templates by the ionChef system (Thermo Fisher Scientific), and the templates were sequenced to a target depth of approximately 15–20 using ionProton (Thermo Fisher Scientific) according to the standard protocol. The version of the semiconductor chip for ionProton used in each sample is given in Supplementary Table S4.

### Bioinformatics analysis and confirmation

Small variants were searched with variantCaller (https://github.com/iontorrent/TS/tree/master/plugin/variantCaller), and large variants were detected using our original program (see supplementary document). Briefly, the reads with clipping sequences were extracted from the BAM data^54^ and realigned to the *C. elegans* genome to obtain the “split reads”^55^. The split reads were classified based on the pattern of break sites and filtered according to the read count. By combining split read analysis and copy number analysis^56^ of variant candidate regions, we detected deletions, duplications, multiplications, insertions, inversions, and translocations. For both the small and large variants, we excluded background variants that were commonly detected in multiple samples. For some of the large variants detected, to confirm that the mutations were indeed present, we performed PCR and Sanger sequencing. Then, we isolated living worms carrying the variants to establish new gene mutant strains. Information on whether a detected variant was confirmed and isolated is provided in Supplementary Tables S5 and S9.

### Induction of the dpy-3 deletion by CRISPR/Cas9

Plasmid solutions containing 100 ng/µl Peft-3::Cas9_dpy-3 sgRNA^48^ and 15 ng/µl Pmyo-2::venus were injected into adult worms. Then, the worms were incubated at 20 °C for 2 or 3 days, and F1 worms that expressed venus protein in the pharynx were transferred to new plates. From each plate, F2 dumpy worms were isolated as new *dpy-3* alleles. The isolated *dpy-3* alleles were sequenced to identify the size of deletions using the primers listed in Supplementary Table S7. Primers were designed to cover the regions from 32 kb upstream to 41 kb downstream of the *dpy-3* gene. In some cases, we were not able to determine the sequence of the breakpoint, and these cases are indicated as “Not identified” in Supplementary Table S8.

### Data analysis

We performed a statistical analysis using R^57^. For the normality test, we used the Shapiro-Wilks test. The chi-squared test, ANOVA and Kruskal–Wallis test were performed using the standard functions. The post hoc Dunnett’s test, Steel–Dwass’s test and Steel’s test were performed using the scripts (http://aoki2.si.gunma-u.ac.jp/R/src). All bar and pie charts were drawn in Excel (Microsoft), and the box-and-whisker plots were drawn in R^57^.

### Machine learning

The learning and prediction process was performed using the scikit-learn library^58^ on Google Colaboratory. The absolute positions of variants, logarithm of deletion and insertion length, copy number ratio, difference of normalized depth between control and samples (supplementary document), frequency of variants, the total number of split reads, and the bias of the positive and negative strand split read count are used as explanatory variables. The objective variable was set to 1 for candidate deletions that could be confirmed by PCR and 0 for those that could not. As training data, we used the variants listed in Supplementary Table S5, and the trained model was tested using the variants listed in Supplementary Table S9. The notebook including the source code was uploaded to GitHub (https://github.com/YujiSue/python/blob/master/DeletionFilter.ipynb).

### Data access

The 889 mutant strains of all the isolated and confirmed variants have already been made available on the NBRP website (https://shigen.nig.ac.jp/c.elegans/mutants/index.xhtml), and their positions can be confirmed on the site (http://rx93.php.xdomain.jp/wormmut.php?lang=EN). The WGS data generated in this study have been submitted to the NCBI BioProject database (http://www.ncbi.nlm.nih.gov/bioproject/) under accession number PRJNA541046.

### Computer code

All the script and programming source codes made for this study have been uploaded to GitHub (https://github.com/YujiSue/YujiSue.github.io). Instructions for installation and use of these applications are provided on each repository site. To generate Supplementary Fig. 1, we made and used GeneMapSVG (version 1.1, https://github.com/YujiSue/BioInfoTools/tree/master/GeneMapSVG). For measuring DNA concentration of WGS library, we made and used an ImageJ^59^ plugin (https://github.com/YujiSue/IJPlugIns/blob/master/Library_Measure.java). For the variant detection and gene annotation using WGS data, we made and used Sutoku (version 1.1, https://github.com/YujiSue/Sutoku). To use these programs, we modulated the format of genomic sequence and gene information obtained from the WormBase^43^ (WS252, ftp://ftp.wormbase.org/pub/wormbase/) using our original codes named as GenomeConverter (version 1.2, https://github.com/YujiSue/BioInfoTools/tree/master/GenomeConverter) and AnnotDBMaker (version 1.1, https://github.com/YujiSue/BioInfoTools/tree/master/AnnotDBMaker), respectively. To confirm the sites of the detected variants and deletion sites in *dpy-3* gene generated by CRISPR by Sanger sequencing, we made and used the VariantDetect (version 1.1, https://github.com/YujiSue/BioInfoTools/tree/master/VariantDetect).

## Supplementary Information

Supplementary Information.

Supplementary Table 1.

Supplementary Table 2.

Supplementary Table 3.

Supplementary Table 4.

Supplementary Table 5.

Supplementary Table 6.

Supplementary Table 7.

Supplementary Table 8.

Supplementary Table 9.
