# Supplementary material for: Efficient collection of a large number of mutations by mutagenesis of DNA damage response defective animals
Source: Sci Rep. 2021 Apr 7;11:7630. doi: 10.1038/s41598-021-87226-7 (PMC8027614; doi:10.1038/s41598-021-87226-7)
Supplement: Supplementary file 1 — Supplementary Information. [file 41598_2021_87226_MOESM1_ESM.pdf]

Efficient collection of a large number of mutations by mutagenesis of DNA damage response defective animals

Yuji Suehiro, Sawako Yoshina, Tomoko Motohashi, Satoru Iwata, Katsufumi Dejima, Shohei Mitani

Supplementary Methods

Quantitative analysis of *pcn-1* gene expression

The expression levels of *pcn-1* were quantified by qPCR using an Applied Biosystems 7500 system. Amplification was performed using the following primers, pcn-1_F1:5'-CGATACCTGCATGCTGAAGTATG-3', pcn-1_F2:5'-CATCAACCTCGGACTTTCGT-3', pcn-1_F3. 5'-CGT TGGTTTCGCTCAAGTTGGAGGTCGG-3', pcn-1_R1:5'-TCGCTGTCAATGTCCATCAT-3', pcn-1_R2:5'-CGCCTTCTGTTTTCCTCATACTTCA-3'. The relative expression levels of *pcn-1* and *aman-1* were determined by the Ct method. Fragments of *aman-1* were amplified by 5'-AGATGGACCTCACCGACAAC-3' and 5'-CTGCAGATTACACGGAAGCA-3'.

Customized protocols to synthesize library DNA for WGS

The library DNA was synthesized using 25-100 ng *C.elegans* genome by the

Library Builder system (Thermo Fisher Scientific). To use this system, the library length to be synthesized, the DNA shear time, and the amount of AMPure XP Reagent (Beckman Coulter) were adjusted to suit the samples. The values for each parameter are listed in Table S4. The obtained library DNA was amplified with the primers 5'-CCACTACGCCTCCGCTTTCCTCTCTATG-3' and 5'-CCATCTCATCCCTGCGTGTC-3' using Platinum PCR SuperMix, High Fidelity (Thermo Fisher Scientific). Then, the amplified fragments were electrophoresed with standard DNA, the concentration of which have already been measured by Agilent 2100 Bioanalyzer, on 2% E-Gel EX Agarose Gels (Thermo Fisher Scientific). Then, the concentrations of them were calculated based on the fluorescent intensity using ImageJ (NIH).

Detection of variants by WGS

The raw sequence reads were mapped to the *C.elegans* genome (WS248) using TMAP (https://github.com/iontorrent/TS/tree/master/Analysis/TMAP)

and the results were obtained as BAM format data. From the BAM files, we extracted the reads with clipped regions and remapped them. For the re-mapping, we divided the clipped sequence into short seed sequences, built a trie, searched for the regions matched to the seed sequence, and expand the seed to make alignment[1]. Based on the alignment scores at all the candidate regions, to which a clipped sequence can be remapped, we selected re-mapped site with a maximum score and constructed a split read (SR) from the re-mapped and pre-mapped sites. The ratio of the score of a SR to the total of all scores calculated when remapping was defined as an error probability (SRerr). The SRs were classified into the five types, deletion, multiplication, inversion, translocation and inverted translocation depending on the chromosomes, strand, and positions of the discrete regions of each SR (Fig.S4B-J).

The SRs were defined as identical if two SRs satisfying the following three criteria. 1) The chromosome and direction are identical. 2) The differences in start positions are less than 50 bases. 3) The difference of variant size is less

than 100 bases. Then, the SRs that were determined to be identical were then merged and their total counts were tallied. The deletion and multiplication type SRs are directly used as the candidates of deletion (Fig.4B) and multiplication (Fig.4C), respectively. By pairing of deletion and insertion type SRs, candidates of deletion with insertion were obtained (Fig.S4D). In the same way, by pairing of inversion and/or translocation type SRs, local inversions (Fig.S4E) or inverted insertions (Fig.S4F), translocation (Fig.S4G), translocational insertion (Fig.S4H), inverted translocation (Fig.S4I) and inverted translocational insertion (Fig.S4J) candidates were obtained. When the total count of SRs was less than 2 for deletion candidates or 3 for the other variant candidates, the variant was ignored. We also cut off the variants with low quality value, defined as Phred's score of SRerr, less than 0.1. From the variant candidates based on the analysis using SRs, we selected final candidates by combining copy number analysis. We calculated the ratio of depth at each base to average depth as the normalized depth (ND). Then, to distinguish whether a deletion

is a homo- or heterozygous, we calculated the ratio of the ND at the deletion site in a sample data to that in a control data, which is obtained by sequencing of non-TMP/UV treatment N2 wild type worms as copy number (CN) ratio (Fig.S4K). When the depth value at the deletion sites in the control data was more than 3.0, we selected variants, of which the CN ratio was less than 0.35 or 0.75, as homo- or heterozygous deletions, respectively. Conversely, if the CN ratio was more than 1.75 or 2.5, we defined them as duplications or multiplications, respectively. In addition, the frequency of variants was calculated by dividing the total number of SRs by the average depth adjacent to the break sites. For variants other than deletions, we then defined a variant as homozygous if its frequency was greater than 0.3. Small variants were detected with the VariantCaller (https://github.com/iontorrent/TS/tree/master/plugin/variantCaller).

We created a list of background variants by integrating the variants that were detected from at least 2 strains, and the background variants were removed from each dataset. Then, only homozygous variants were selected

(Table S5).

Induction of the *dpy-3* deletion by CRISPR/Cas9

Plasmid solutions containing 100 ng/µl Peft-3::Cas9_dpy-3 sgRNA (Dejima et al. 2018; Iwata et al. 2016), and 15 ng/µl Pmyo-2::venus were injected into adult worms. Then, the worms were incubated at 20 °C for 2 or 3 days, and F1 worms that express venus protein in the pharynx were transferred to new plates. From each plate, F2 dumpy worms were isolated as new dpy-3 alleles. The isolated dpy-3 alleles were sequenced to identify the size of deletions using the primers listed in Table S7. Primers were designed to cover the regions from 32 kb upstream to 41 kb downstream of the *dpy-3* gene. In some cases, we were not able to determine the sequence of the breakpoint, and the number of these cases is indicated as "Not identified" in Table S8.

Supplementary Reference

1. Aho, Alfred V., and Margaret J. Corasick. 1975. "Efficient String Matching: An Aid to

Bibliographic Search." Communications of the ACM 18(6):333–40.

Supplementary Figure Legends

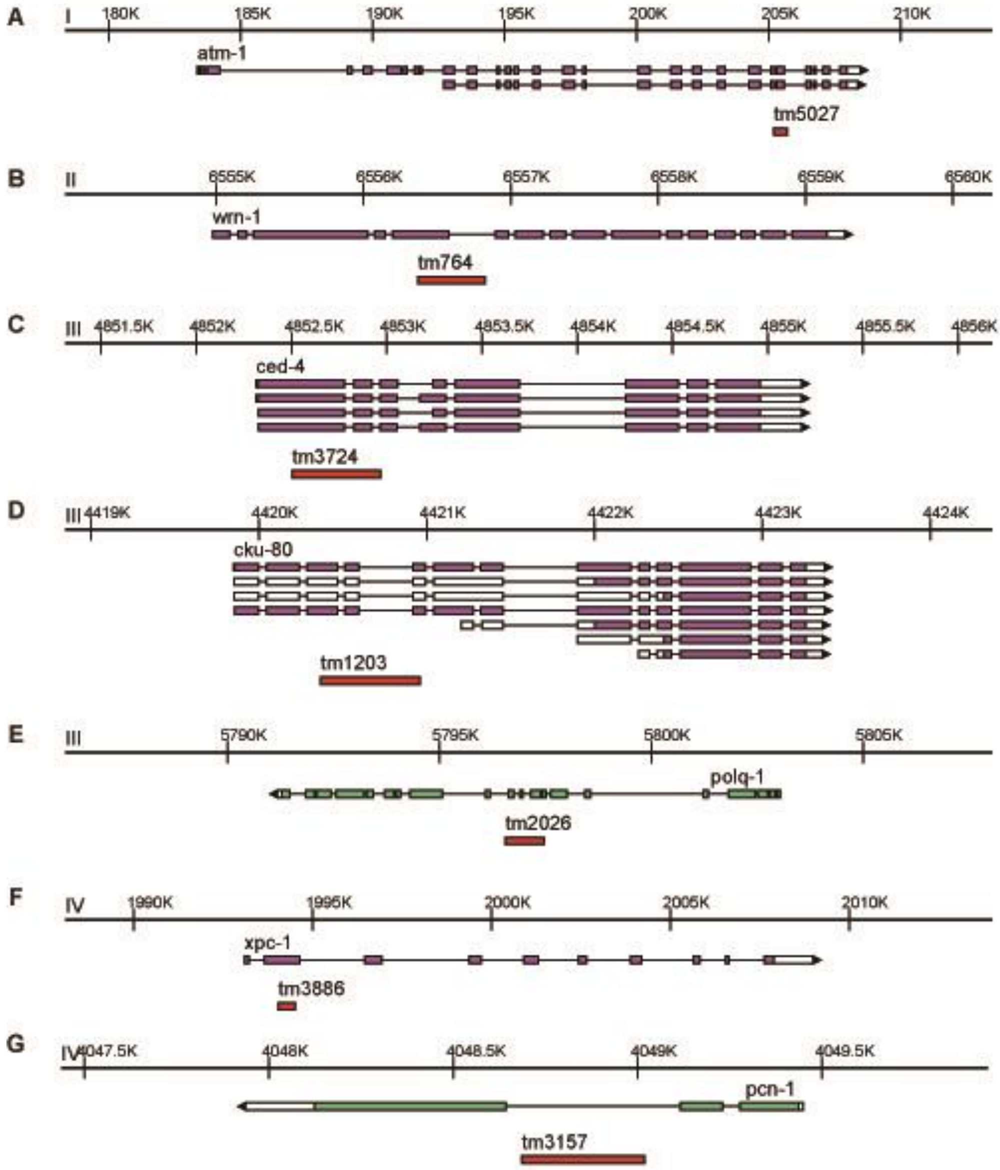

Fig.S1

Figure S1 Positions of genes and mutant alleles used in this study

(A-G) The positions of mutations and DDR related genes *atm-1* (A), *wrn-1* (B), *ced-4* (C), *cku-80* (D), *polq-1* (E), *xpc-1* (F) and *pcn-1* (G) were displayed. The site of deletions (red), UTR (white) and CDS (magenta or green) are

exhibited as boxes. Triangles show the gene orientation. The figures were drawn using our program (https://github.com/YujiSue/BioInfoTools/tree/master/GeneMapSVG).

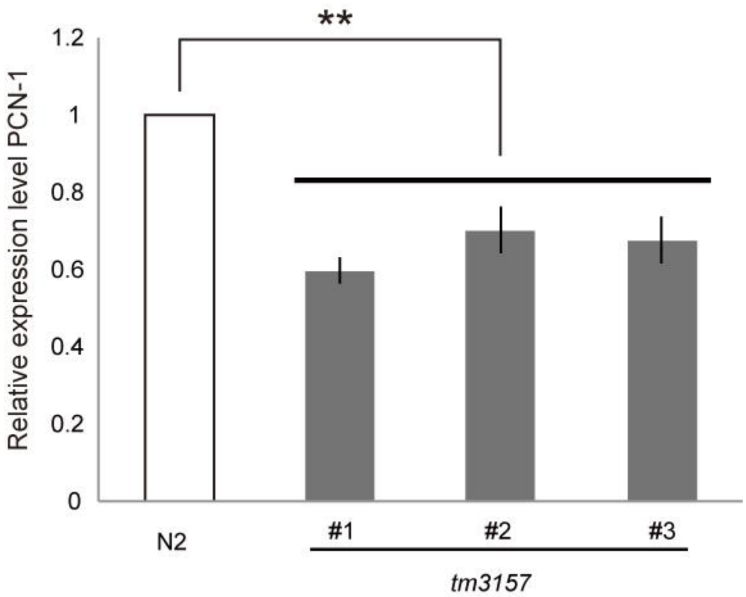

Figure S2 Quantitative analysis of *pcn-1* expression level

The expression level of pcn-1 by qPCR.

The relative expression levels were measured by ΔΔCt method. The amplification was performed using three pairs of primers and each relative expression level was displayed as gray bars. #1: pcn-1_F1 and pcn-1_R1, #2: pcn-1_F2 and pcn-1_R2, #3: pcn-1_F3 and pcn-1_R1. The sequences of each primer were described in the supplementary methods. ** $p < 0.01$ Dunnett's test (n=4).

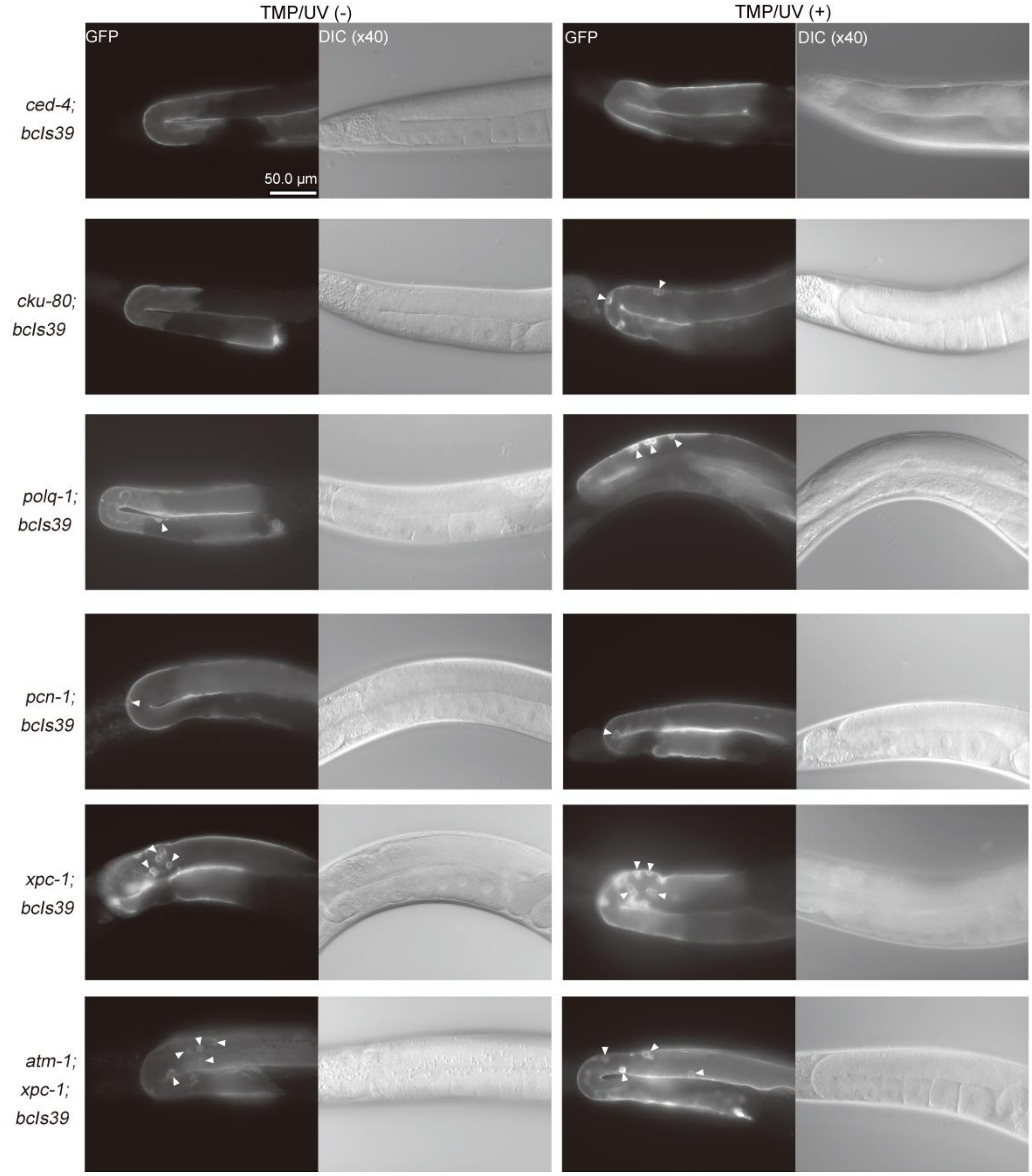

Figure S3 Representative images of germ cell apoptosis in DDR mutants.

Untreated control (left panel) and TMP/UV treated worms (right panel) derived from each DDR mutant were displayed. White arrowheads indicate the apoptotic cells.

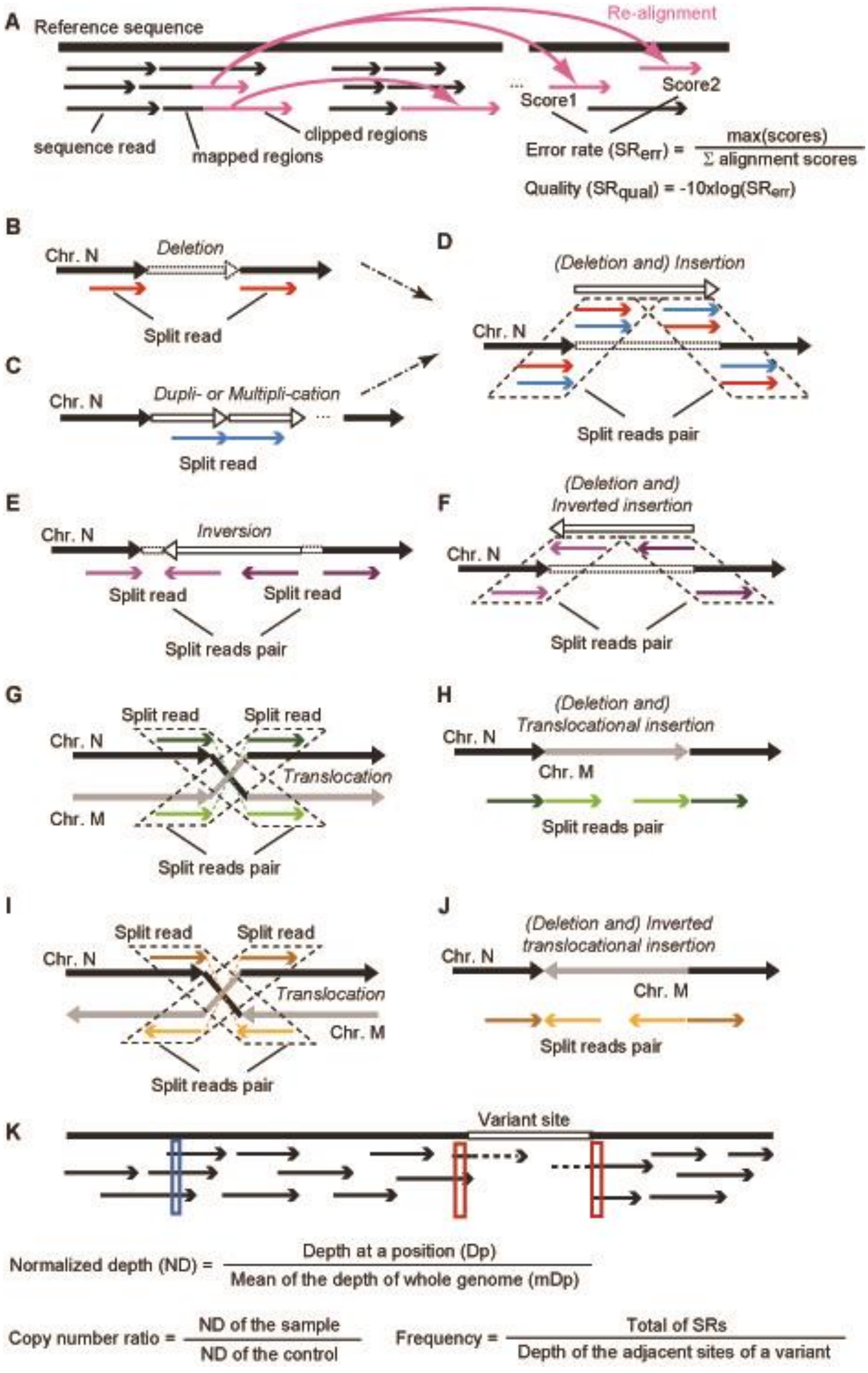
A
Reference sequence
Re-alignment
Score2
Score1
sequence read
mapped regions
clipped regions
Error rate (SRerr) = max(scores) / Σ alignment scores
Quality (SRqual) = -10xlog(SRerr)
B
Chr. N
Deletion
Split read
C
Chr. N
Dupli- or Multipli-cation
Split read
D
(Deletion and) Insertion
Chr. N
Split reads pair
E
Chr. N
Inversion
Split read
Split read
Split reads pair
F
(Deletion and)
Inverted insertion
Chr. N
Split reads pair
G
Split read
Split read
Chr. N
Translocation
Chr. M
Split reads pair
H
(Deletion and)
Translocational insertion
Chr. N
Chr. M
Split reads pair
I
Split read
Split read
Chr. N
Translocation
Chr. M
Split reads pair
J
(Deletion and) Inverted
translocational insertion
Chr. N
Chr. M
Split reads pair
K
Variant site
Normalized depth (ND) = Depth at a position (Dp) / Mean of the depth of whole genome (mDp)
Copy number ratio = ND of the sample / ND of the control
Frequency = Total of SRs / Depth of the adjacent sites of a variant

Figure S4 Schemes to detect large deletions and structure variants.

(A) After the primary mapping using TMAP, clipped sequences were re-mapped to *C. elegans* genome. When re-aligning, we calculated the alignment scores to the candidate mapping regions and created SRs with the highest scoring locations as the most likely candidates. The ratio of the maximum score to all the calculated scores was used as the alignment error (SR_{err}), and the Phred's score was used as the quality value. (B-J) Six variants were defined based on the type of SRs: if the anterior and posterior regions of the SRs are at discrete sites on a single chromosome, they are classified as candidates for deletion (B) or duplication/multiplexing (C). If the two SRs are on the same chromosome, they can be classified as insertional deletions (D) if two regions of each SR are aligned in the same direction, or inversions (E, F) if they are aligned in the opposite direction. Based on the site of inversion-type SRs, the local inversion (E) or inverted insertion (F) are classified. A SR consisted of regions mapped to different chromosomes is defined as the translocation type. If two regions of the SR are aligned to the

opposite strand, the SR is defined as the inverted translocation. By pairing those SRs, translocation (G), translocational insertion (H), inverted translocation (I), and inverted translocational insertion (J) are classified. (K) The normalized depth (ND) was calculated from the depth at each position and the averaged depth. Then the copy number ratio was calculated from the ND of a sample to that of the control, which is a merged data of non-TMP/UV treated wild type animals. The frequency of a variant was defined as the ratio of the total count of SRs and the averaged depth adjacent to the variant.

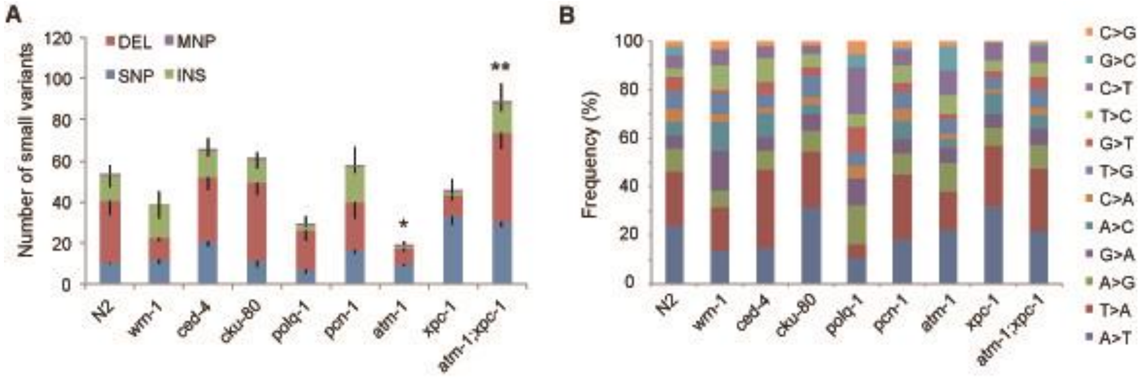

Figure S5 Small variants detected in NGS analysis.

Sequenced data analyzed in Figure 3 were used for the detection of small variants. (A) The mean number of single/multiple nucleotide variants (blue/violet), short deletions (red) and insertions (green) are displayed as a bar graph. The total count was significantly decreased in *atm-1* and increased in *atm-1;xpc-1* compared to the wild type (p-values were 0.014 and 0.0018, respectively. Dunnett's test). (B) The frequency of each single nucleotide substitution was shown.

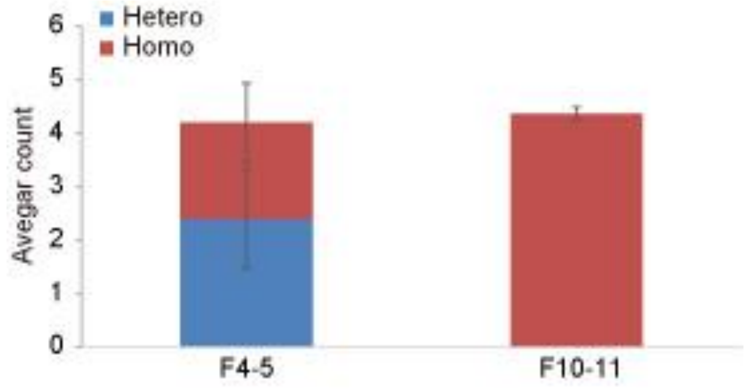

Figure S6 The number of variants detected by WGS between generations

The hetero (blue bars) and homozygous (red bars) variants derived from F4-5 and F10 of *atm-1;xpc-1* mutants are shown. All variants are summarized in Table S6. There was no significant difference between the generations in the total counts (p-value was 0.86, Student's t-test).

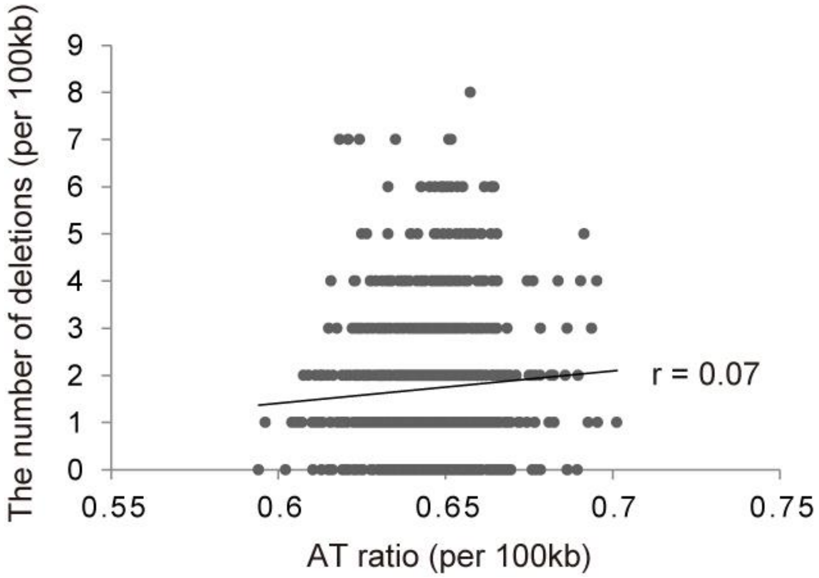

Figure S7 Bias of variants positions

The correlation between number of deletions and A/T ratios per 100kb. Pearson's correlation value was 0.07. The plot image was generated using R software.
